# Long-Term Effects of Ketoanalogues on Mortality and Renal Outcomes in Advanced Chronic Kidney Disease Patients Receiving a Low-Protein Diet

**DOI:** 10.3390/nu12092708

**Published:** 2020-09-04

**Authors:** Yi-Chun Wang, Shu-Hui Juan, Chu-Lin Chou, Tsung-Cheng Hsieh, Jung-Lun Wu, Te-Chao Fang

**Affiliations:** 1 Division of Nephrology, Taipei Tzu Chi Hospital, Buddhist Tzu Chi Medical Foundation, New Taipei City 231, Taiwan; m92chung@tzuchi.com.tw; 2 School of Medicine, Tzu Chi University, Hualien 970, Taiwan; 3 Department of Physiology, School of Medicine, College of Medicine, Taipei Medical University, Taipei 110, Taiwan; juansh@tmu.edu.tw; 4 Graduate Institute of Medical Sciences, College of Medicine, Taipei Medical University, Taipei 110, Taiwan; 5 Division of Nephrology, Department of Internal Medicine, Hsin Kuo Min Hospital, Taipei Medical University, Taoyuan City 320, Taiwan; chulin.chou@gmail.com; 6 Division of Nephrology, Department of Internal Medicine, School of Medicine, College of Medicine, Taipei Medical University, Taipei 110, Taiwan; 7 TMU Research Center of Urology and Kidney, Taipei Medical University, Taipei 110, Taiwan; 8 Division of Nephrology, Department of Internal Medicine, Tri-Service General Hospital, National Defense Medical Center, Taipei 114, Taiwan; 9 Graduate Institute of Medical Sciences, Tzu Chi University, Hualien 970, Taiwan; tchsieh@mail.tcu.edu.tw; 10 Department of Occupational Medicine, Hualien Tzu Chi Hospital, Buddhist Tzu Chi Medical Foundation, Hualien 970, Taiwan; alun0311@gmail.com; 11 Division of Nephrology, Department of Internal Medicine, Taipei Medical University Hospital, Taipei Medical University, Taipei 110, Taiwan

**Keywords:** ketoanalogues, advanced chronic kidney disease, low-protein diet

## Abstract

The effects of ketoanalogues (KA) supplementation on mortality and progression to dialysis in patients with pre-dialysis stage 5 chronic kidney disease (CKD) receiving a low-protein diet (LPD) remain ambiguous. From Taiwan’s National Health Insurance Research Database during 1996–2011, 165 patients with pre-dialysis CKD on an LPD (0.6 g/kg/day) with KA supplementation were matched with 165 patients with pre-dialysis CKD on an LPD without KA supplementation. Of the 165 patients with advanced CKD receiving KA supplementation, 34 (20.6%) died, and 124 (75.2%) underwent long-term dialysis during the study period. There was no significant difference in mortality between the KA-user group and the KA-nonuser group (adjusted hazard ratio [HR], 1.41; 95% confidence interval [CI], 0.68–2.93; *p* = 0.355). KA supplementation significantly increased long-term dialysis risk (adjusted HR, 1.41; 95% CI, 1.04–1.90; *p* = 0.025) and combined outcome risk (defined as long-term dialysis and death; adjusted HR, 1.37; 95% CI, 1.02–1.83; *p* = 0.034). KA supplementation also increased long-term dialysis risk (adjusted HR, 1.49; 95% CI, 1.00–2.20; *p* = 0.048) in the subgroup of pre-dialysis patients with diabetes mellitus (DM), but not in those patients without DM. In conclusion, KA supplementation might increase long-term dialysis risk in patients with advanced CKD receiving an LPD, but it did not increase mortality.

## 1. Introduction

A low-protein diet (LPD) has been found to delay chronic kidney disease (CKD) progression in studies on animal and carefully selected patients with CKD [1,2,3,4]. In the systematic review and meta-analysis of randomized controlled trials, a protein-restricted diet may reduce kidney function deterioration rate and renal failure risk of patients with CKD, but did not produce a positive effect on all-cause death events [5]. However, considering non-diabetic patients with CKD 4 and 5, a recent systematic review and meta-analysis of randomized controlled trials showed that compared with a normal-protein diet, an LPD may make little difference in the rate of decreasing renal function in non-diabetic patients with CKD 4 and 5 who progress to renal replacement therapy, but a very low protein diet (VLPD) probably decrease the number of non-diabetic patients with CKD 4 and 5, who progress to renal replacement therapy [6].

Studies have demonstrated that the benefits of ketoanalogues (KA) supplementation on mortality and renal outcomes in CKD patients undergoing dietary protein restriction are controversial [7,8,9,10,11]. First, regarding the effects of KA supplementation through a VLPD on patients with stage 4 and 5 CKD, Garneata et al. conducted a prospective randomized controlled trial in 207 patients with estimated glomerular filtration rate (eGFR) < 30 mL/min and noted that KA supplementation with a vegetarian VLPD (0.3 g/kg body weight/day) could defer dialysis initiation compared with an LPD (0.6 g/kg body weight/day) alone [7]. However, the effects of KA supplementation with an LPD on patients with stage 5 CKD were inconclusive [8,9,10,11]. A study with only 45 patients reported no significant difference in the rate of decline of renal function between non-dialytic stage 5 CKD patients with an LPD (0.6–0.8 g/kg/day) plus KA supplementation for six months and those with an LPD alone [8]. By contrast, Wu et al. showed that KA supplementation reduced dialysis and mortality risks in patients with anemic advanced CKD [9]. However, this study design has raised concerns of immortal time bias because authors compared KA use periods and their countermanded periods of KA use of the same individuals as a period without treatment of KA. In the Modification of Diet in Renal Disease (MDRD) Study, this largest study concerning KA showed that the protein-restricted diet only slightly lessened the decline in GFR, due to the small advantage caused by the protein restriction, not the KA supplementation [11]. Moreover, for long–term follow-up, the observation in the MDRD study suggested an increase in the risk of death, due to a problem of LPD-related malnutrition [12]. Thus, the clarifications for the differences between the results of studies investigated to evaluate KA and LPD are of particular interest.

The meta-analysis displayed that an insignificant effect of KA supplementation was found in preserving renal function [13,14]. Besides, according to a recent meta-analysis, a subgroup analysis of a restricted protein diet showed that an LPD with KA supplementation could prevent renal function deterioration compared with LPD alone in patients with eGFR >18 mL/min/1.73 m^2^, but there was no data from patients with eGFR <18 mL/min/1.73 m^2^. Because the efficacy of protein-restricted regimens remained unclear [10], the feasibility and compliance of patients to the LPD were repeatedly questioned and reported as poorly obeyed, and the risk of malnutrition was frequently raised [15,16,17,18]. Therefore, we conducted this study to evaluate the long-term effects of KA on mortality and renal outcomes in patients with pre-dialysis stage 5 CKD (advanced CKD) who received an LPD.

## 2. Materials and Methods

### 2.1. Data Collection

Data on the management of patients with advanced CKD from January 1996 to December 2011 were extracted from Taiwan’s Longitudinal Health Insurance Database (LHID). The LHID holds all of the registration files and details about the original claims that relate to 1 million beneficiaries from the National Health Insurance (NHI) Research Database (NHIRD) for research purposes. NHIRD holds information relating to outpatient data, inpatient data, disease profiles, drugs prescribed, intervention procedures, and medical costs for >99% of the population in Taiwan, which equates to >22 million people. NHIRD database is one of the highest quality databases worldwide and is widely used for longitudinal cohort studies, including our previous reports [19,20,21,22,23,24,25,26,27]. The diagnosis codes are based on the International Classification of Diseases, Ninth Revision. To protect privacy, individuals’ identifications are encrypted within the NHI database. This study was exempted from review by the Taipei Tzu Chi Hospital Review Board (Protocol number: 03-W02-091).

### 2.2. Study Population

**Figure 1:**
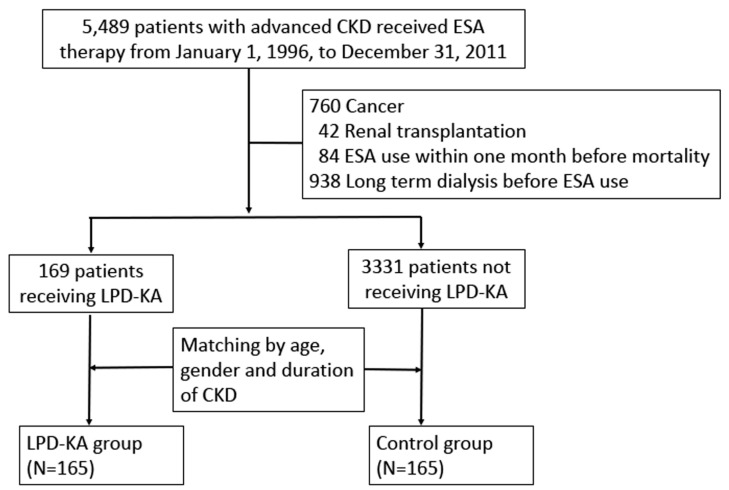
Patient selection flow. CKD = chronic kidney disease; ESA = erythropoiesis-stimulating agent; KA = ketoanalogues; LPD = low-protein diet.

This was a population-based, longitudinal cohort study. The study subject selection process is illustrated in Figure 1. According to NHI reimbursement regulations, patients with CKD are treated with erythropoiesis-stimulating agents (ESAs) if their serum creatinine levels are >6 mg/dL (pre-dialysis stage 5 CKD) and their hematocrit levels are <28%; hence, we identified patients with advanced CKD as those receiving ESA treatment. In Taiwan, patients with CKD who were on an LPD with serum creatinine levels above 6 mg/dL for three consecutive months could also receive KA supplementation (Ketosteril, Fresenius Kabi, Bad Homburg, Germany) without a copayment, with a maximum total dose of six tablets daily. The timing of the start of advanced CKD follow-up was defined as the first time when ESA was administered. The study’s exclusion criteria were cancer diagnosis, renal transplantation, ESA uses within one month before mortality, and long-term dialysis before the first ESA dose was administered. Patients with advanced CKD who received KA during the study period were enrolled in the KA-user group. The KA-nonuser group was matched 1:1 to the KA-user group by age, sex, and the duration of advanced CKD. Both the groups were administered an LPD (0.6 g/kg/day) as recommended in the Kidney Disease Outcomes Quality Initiative guidelines.14 We also performed a subgroup analysis of CKD patients with and without diabetes mellitus (DM).

### 2.3. Covariates

Baseline demographic and clinical characteristics recorded before the index date were obtained. ICD-9-CM disease diagnostic codes for previous or coexisting diseases and Anatomical Therapeutic Chemical (ATC) codes for medication are listed in Table 1. The comorbidities and prescribed medication were as the follows: Congestive heart failure, peripheral vascular disease, cerebrovascular disease, dementia, chronic obstructive pulmonary disease (COPD), rheumatic disease, peptic ulcer disease, diabetes mellitus (DM), hemiplegia, moderate and severe liver disease, and angiotensin-converting enzyme inhibitors/angiotensin receptor blockers (ACEIs/ARBs). The ICD-9-CM codes of Charlson comorbidity index were as presented in Supplementary Table S1.

### 2.4. Outcome Measurements and Definitions

The primary outcomes were renal outcome and mortality. The renal outcome was defined as the date on which long-term dialysis for at least 90 days began. The observation period began from the date on which ESA usage started, and it ended at death, on the start date for long-term dialysis, or on 31 December 2011. The rates of death and long-term dialysis were expressed in terms of numbers per 1000 patient-years.

### 2.5. Statistical Analysis

The patients’ baseline characteristics were compared using a two-sided *t*-test and χ2 test. A Cox proportional hazards regression analysis was used to evaluate the effects of variables on mortality and renal outcome. All statistical tests were two-sided, and a *p* of <0.05 was considered statistically significant. All the statistical analyses were performed using SAS for Windows (version 9.3; SAS Institute, Inc., Cary, NC, USA).

## 3. Results

We enrolled 5849 patients with advanced CKD in the present study. Of these, 1824 patients were excluded because of cancer, renal transplantations, long-term dialysis, and mortality before the ESA treatment began. After the patients were matched by age, sex, and advanced CKD duration, 165 patients were enrolled in the KA-user group (Figure 1). The mean age of the patients in the KA-user group was 57.8 years, and this group comprised 44.9% of men. The KA-user group had a significantly lower Charlson comorbidity score and lower percentages of patients with peripheral vascular disease, cerebrovascular disease, and DM than the KA-nonuser group. A subgroup analysis of advanced CKD patients with and without DM revealed no significant differences in relation to age, sex, or Charlson comorbidity score; in relation to the presence of congestive heart failure, peripheral vascular disease, cerebrovascular disease, dementia, chronic obstructive pulmonary disease, rheumatic disease, peptic ulcer disease, hemiplegia, or moderate and severe liver disease; or in relation to angiotensin-converting enzyme inhibitor and angiotensin II receptor usage between the KA-user and KA-nonuser groups (Table 2).

The analysis of mortality and renal outcomes in patients with advanced CKD using Cox proportional hazards regression analysis is presented in Table 3. Among the patients with advanced CKD receiving KA, 34 patients (20.6%) died, and 124 patients (75.2%) underwent long-term dialysis during the study period. The mean period outcomes of death and long-term dialysis were 15 and 19 months in the KA-user and KA-nonuser groups, respectively. The mortality rate was 58.96 per 1000 patient-years, and the long-term dialysis rate was 696.97 per 1000 patient-years in the KA-user group. No significant difference was observed between the KA-user and KA-nonuser groups with respect to mortality (adjusted hazard ratio [HR], 1.41; 95% confidence interval [CI], 0.68–2.93; *p* = 0.355). Patients with advanced CKD who were treated with KA had a significantly higher risk of long-term dialysis (adjusted HR, 1.41; 95% confidence interval [CI], 1.04–1.90; *p* = 0.025) and a significantly higher risk of combined outcomes, such as long-term dialysis and death (adjusted HR, 1.37; 95% CI, 1.02–1.83; *p* = 0.034), than those who were not treated with KA. KA treatment increased the risk of long-term dialysis in the subgroup of advanced CKD patients with DM (adjusted HR, 1.49; 95% CI, 1.00–2.20; *p* = 0.048), but not advanced CKD patients without DM.

**Figure 2:**
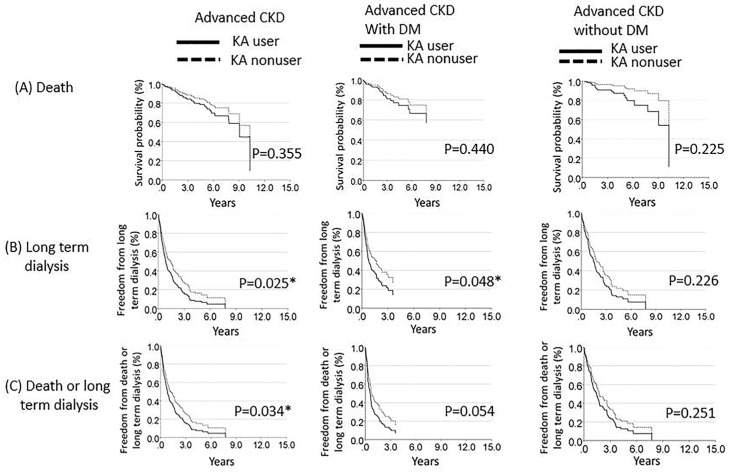
Survival curves, derived from the Cox proportional hazards regression analysis, for (**A**) death, (**B**) long-term dialysis, and (**C**) the combined outcomes defined as death or long-term dialysis, for advanced chronic kidney disease patients who received or did not receive keto/amino acid treatment. CKD, chronic kidney disease; DM, diabetes mellitus; KA, ketoanalogues. * Significant *p* (*p* < 0.05)

The survival curves for death, long-term dialysis, and the combined outcomes of death and long-term dialysis in patients with advanced CKD, were derived from the Cox proportional hazards regression analysis (Figure 2). The differences between the survival curves for the KA-user and KA-nonuser groups were significant with respect to long-term dialysis (*p* = 0.025) and the combined outcomes of death and long-term dialysis (*p* = 0.034). These findings indicate that the KA-user group was at a higher risk of requiring long-term dialysis and combined outcomes than the KA-nonuser group. The subgroup analysis of the advanced CKD patients with DM indicated that the KA-user group had significantly worse outcomes in relation to long-term dialysis compared with the KA-nonuser group (*p* = 0.048).

## 4. Discussion

The main findings of this study are that an LPD with KA supplementation might increase the risk of long-term dialysis in patients with advanced CKD, and this risk was obvious in the subgroup of advanced CKD patients with DM, but not in advanced CKD patients without DM.

The effects of KA on patients with advanced CKD with an LPD are unclear [8,9,10,11]. Wu et al. conducted a retrospective cohort study in which 1483 anemic patients with advanced CKD receiving an LPD (0.8 g/kg/day) plus KA for 180 days were included [9]. Their result showed that KA supplementation reduced dialysis and mortality risks in anemic patients with advanced CKD. However, this study design has raised concerns of immortal time bias because they compared KA use periods and their countermanded KA use periods of the same individual as a period without KA treatment. Additionally, their result was not supported by a recent meta-analysis study conducted by Li et al., who examined the effects of KA on CKD deterioration [10]. Four studies (394 patients) were included in the subgroup of eGFR <18 mL/min per 1.73 m^2^, and four studies (267 patients) were included in the subgroup of eGFR >18 mL/min/1.73 m^2^. KA supplementation benefits patients with CKD with eGFR >18 mL/min/1.73 m^2^ (mean difference [MD], 5.81; 95% CI, 3.19, 8.44; *p* < 0.0001), but not with eGFR <18 mL/min/1.73 m^2^ (MD, 1.87; 95% CI, −0.08, 3.81; *p* = 0.06). Furthermore, a subgroup analysis of a restricted protein diet was performed, and it revealed that an LPD plus KA could prevent renal function deterioration compared with an LPD alone with the eGFR >18 mL/min/1.73 m^2^, but there was no data from patients with eGFR <18 mL/min/1.73 m^2^. Our results indicated that KA supplementation might increase the risk of long-term dialysis in patients with advanced CKD with an LPD. Therefore, a randomized control study is required to clarify the renal protective effects of KA on patients with advanced CKD with an LPD compared with those of LPD alone.

Additionally, a randomized controlled trial reported that a KA-supplemented VLPD could benefit dialysis initiation in patients with stage 4 and stage 5 CKD [7]. Nevertheless, our result showed that KA supplementation could not offer renal protection in patients with advanced CKD with an LPD. The reasons for the worse outcomes of a KA supplementation on patients with advanced CKD with an LPD are unclear. However, this might be related to its administration without adjusting other protein intakes, which lead to nutrition-related comorbidities, such as metabolic acidosis and protein-energy wasting in those patients [10]. A study conducted by Huang et al. also suggested that total protein intake in the KA-user group was higher than that in the KA-nonuser group, and the higher total nitrogen load leads to adverse outcomes [28]. Therefore, assessments by experienced dietitians are crucial for every advanced CKD patient because they can adjust the intake of other proteins in relation to KA supplementation.

The effects of KA on advanced CKD patients with DM have not been examined yet through randomized controlled trials. DM is the leading cause of CKD, and it is the major cause of target organ damage observed in patients with CKD and DM [29]. Lowering protein intakes to 0.6 g/kg/day is recommended for adults who have CKD with and without DM [30]. In this study, KA supplementation increased the risk of long-term dialysis in the subgroup of advanced CKD patients with DM but not in the subgroup of advanced CKD patients without DM. Moreover, advanced CKD patients with DM started long-term dialysis more quickly than advanced CKD patients without DM. Additional studies to clarify the mechanisms underlying the effects of KA supplementation on advanced CKD patients with and without DM are required.

This study has some strengths. The data are from the NHI research database includes most inpatient and outpatient medical practice for Taiwan’s 23 million residents. It is one of the largest databases in the world and has been used in many observational studies [19,20,21,22,23,24]. In this study, we had minimized the selection bias by matching the control group with age, sex, and the duration of advanced CKD. We also adjusted the Charlson comorbidity score and the usage of angiotensin-converting enzyme inhibitor and angiotensin II receptor blocker in the Cox regression model to minimize confounding factors. However, this study has several limitations. First, we could not obtain information from the NHI database about the patients’ body height, body weight, personal habits (physical activity, lifestyle, smoking, and alcohol consumption), nutritional-inflammation status, causes of kidney disease, and laboratory data (creatinine, eGFR, cystatin C, Ca, P, iPTH, Hb, HbA1c) in the NHIRD. Second, we could not evaluate the amount of dietary protein intake and the compliance of LPD and KA in every patient. Third, we did not balance the length of time with diabetes and insulin versus non-insulin. Fourth, we did not balance in terms of diabetes as a cause of the CKD, but we did analysis for the DM subgroup. Fifth, there may have been allocation bias and prescription bias because this study was an observational study for drug epidemiology, not a randomized controlled trial.

## 5. Conclusions

This observational study implied that a KA supplementation on patients with advanced CKD receiving an LPD might carry the risk of long-term dialysis and composite outcomes (long-term dialysis and death). This risk was obvious in the subgroup of advanced CKD patients with DM, but not in advanced CKD patients without DM. A further randomized control study is required to evaluate the renal effects of KA in patients with advanced CKD receiving an LPD.

## Figures and Tables

**Table 1:** Disease diagnosis codes according to the international classification of diseases, ninth revision, clinical modification (ICD-9-CM), and prescribed medications are classified based on anatomical therapeutic chemical (ATC) classification.

Comorbidity/Medication	ICD-9-CM Disease Codes/ATC Codes
Congestive heart failure	428–428.9
Peripheral vascular disease	443.9, 441–441.9, 785.4, V43.4
Cerebrovascular Disease	430–437
Dementia	290–290.9
COPD	490–496, 505, 506.4
Rheumatic disease	710.0, 710.1, 710.4, 714.0–714.2, 714.81, 725
Peptic Ulcer Disease	531–534.9
DM	250–250.3, 250.7
Hemiplegia	342, 438
Moderate or severe liver disease	456–456.21, 572.2–572.8
ACEI/ARB	C09

ACEI = angiotensin-converting enzyme inhibitor; ARB = angiotensin receptor blockers; DM = diabetes mellitus; COPD = chronic obstructive pulmonary disease.

**Table 2:** Patient characteristics.

	Advanced CKD			Advanced CKD with DM			Advanced CKD without DM		
	KA User (*n* = 165)	KA Nonuser (*n* = 165)	*p*	KA User (*n* = 67)	KA Nonuser (*n* = 107)	*p*	KA User (*n* = 98)	KA Nonuser (*n* = 58)	*p*
Age (years)	57.8 ± 12.1	57.8 ± 12.1	0.99	62.0 ± 8.3	59.3 ± 10.2	0.07	55.0 ± 13.5	55.0 ± 14.8	0.99
Gender (male/female)	74/91 (44.9/55.1)	74/91 (44.9/55.1)	-	38/29 (56.7/43.3)	51/56 (47.7/52.3)	0.28	36/62 (36.7/63.3)	23/35 (39.7/60.3)	0.74
Charlson score	6.4 ± 3.0	7.6 ± 3.1	<0.01	4.8 ± 2.0	5.3 ± 2.7	0.13	8.8 ± 2.6	8.9 ± 2.7	0.97
Congestive heart failure	20 (12.1%)	31 (18.8%)	0.09	15 (22.4%)	27 (25.2%)	0.70	5 (5.1%)	4 (6.9%)	0.73
Peripheral vascular disease	4 (2.4%)	14 (8.5%)	<0.01	3 (4.5%)	11 (10.3%)	0.17	1 (1.0%)	3(5.2%)	0.15
Cerebrovascular Disease	23 (13.9%)	41 (24.9%)	0.01	17 (25.4%)	35 (32.7%)	0.30	6 (6.1%)	6 (10.3%)	0.36
Dementia	2 (1.2%)	3 (1.8%)	0.31	1 (1.5%)	3 (2.8%)	0.66	1 (1%)	0 (0%)	1.00
COPD	36 (21.8%)	50 (30.3%)	0.08	21 (31.3%)	34 (31.8%)	0.95	15 (15.3)	16 (27.6%)	0.06
Rheumatic disease	8 (4.8%)	16 (9.7%)	0.09	3 (4.5%)	8 (7.5%)	0.53	5 (5.1%)	8 (13.8%)	0.07
Peptic Ulcer Disease	66 (40.0%)	79 (47.9%)	0.15	31 (46.3%)	58 (54.2%)	0.31	35 (35.7%)	21 (36.2%)	0.95
DM	67 (40.6%)	107 (64.8%)	<0.01			-			-
Hemiplegia	7 (4.2%)	4 (2.4%)	0.36	5 (7.5%)	4 (3.7%)	0.31	2 (2.0%)	0(0)	0.53
Moderate or severe liver disease	0 (0)	1 (0.61%)	-	0 (0%)	0 (0%)	-	0 (0%)	1 (1.7%)	-
ACEI/ARB	156 (94.6)	152 (92.1)	0.38	67 (100.0)	102 (95.3)	0.16	89 (90.8)	50 (86.2)	0.37

ACEI = angiotensin-converting enzyme inhibitor; ARB = angiotensin II receptor block; CKD = chronic kidney disease; DM = Diabetes mellitus; KA = ketoanalogues.

**Table 3:** Cox proportional hazard model for outcomes in patients with advanced CKD.

		Advanced CKD		Advanced CKD with DM		Advanced CKD without DM	
		KA User (*n* = 165)	KA Nonuser (*n* = 165)	KA User (*n* = 67)	KA Nonuser (*n* = 107)	KA User (*n* = 98)	KA Nonuser (*n* = 58)
**Death**	Number (%)	34 (20.6%)	12 (7.3%)	17 (25.4%)	10 (9.3%)	17 (17.3%)	2 (3.4%)
	Incidence rate per 1000 patient-years	58.96	44.64	89.55	63.71	43.95	17.88
	Adjusted HR (95% CI) (KA user vs. KA nonuser)	1.41 (0.68—2.93), *p* = 0.355		1.46 (0.59—3.33), *p* = 0.440		2.69 (0.54—13.31), *p* = 0.225	
Long-term dialysis	Number (%)	124 (75.2%)	83 (50.3%)	49 (73.1%)	57 (53.3%)	75 (76.5%)	26 (44.8%)
	Incidence rate per 1000 patient-years	696.97	486.49	977.67	601.30	586.88	342.95
	Adjusted HR (95% CI) (KA user vs. KA nonuser)	1.41 (1.04—1.90), *p* = 0.025 *		1.49 (1.00—2.20), *p* = 0.048 *		1.35 (0.83—2.20), *p* = 0.226	
Death or long-term dialysis	Number (%)	128 (77.6%)	88 (53.3%)	52 (77.6%)	61 (57.0%)	76 (77.6%)	27 (46.6%)
	Incidence Rate per 1000 patient-years	721.14	521.17	1043.46	652.77	595.32	358.23
	Adjusted HR (95% CI) (KA user vs. KA nonuser)	1.37 (1.02—1.83), *p* = 0.034 *		1.45 (0.99—2.13), *p* = 0.054		1.32 (0.82—2.14), *p* = 0.251	

* Significant *p* (*p* < 0.05). CI = confidence interval; CKD = chronic kidney disease; DM = diabetes mellitus; HR = hazard ratio; KA = ketoanalogues.

## Supplementary Materials

The following are available online at https://www.mdpi.com/2072-6643/12/9/2708/s1, Table S1: Charlson Comorbidity Index.
