# Supplementary material for: Long-Term Effects of Ketoanalogues on Mortality and Renal Outcomes in Advanced Chronic Kidney Disease Patients Receiving a Low-Protein Diet
Source: Nutrients. 2020 Sep 4;12(9):2708. doi: 10.3390/nu12092708 (PMC7551296; doi:10.3390/nu12092708)
Supplement: Supplementary file 1 [file nutrients-12-02708-s001.pdf]

Table S1. Charlson Comorbidity Index.

Comorbid conditions	ICD-9-CM	Weights
Congestive heart failure	428–428.9	1
Peripheral vascular disease	443.9, 441–441.9, 785.4, V43.4	1
Cerebrovascular disease	430–437	1
Rheumatic disease	710.0, 710.1, 710.4, 714.0–714.2, 714.81, 725	1
Peptic ulcer disease	531–534.9	1
Hemiplegia or paraplegia	344.1, 342–342.9	2
Dementia	290–290.9	1
Chronic pulmonary disease	490–496, 505, 506.4	1
Myocardial infarction	410–410.9, 412	1
Renal disease	582–582.9, 583–583.7, 585, 586, 588–588.9	2
Any malignancy	140–172.9, 174–195.8	2
Leukemia	203–208.9	2
Lymphoma	200–202	2
Metastatic solid tumor	196–199.1	6
Mild liver disease	571.2, 571.4–571.49, 571.5, 571.6	1
Moderate or severe liver disease	456–456.21, 572.2–572.8	3
Diabetes without chronic complication	250.0x	2
Diabetes with chronic complication	250.1x–250.9x	2
AIDS	042	6
Age group		
0–49 years		0
50–59 years		1
60–69 years		2
70–79 years		3
80–89 years		4
90–99 years		5

Score defined as the sum of the patient's disease and age weights.
